# Impacts of Combined Microplastics and Heavy Metal Pollution on Aquatic Ecological Health: Insights from Trophic Levels

**DOI:** 10.3390/toxics14100848

**Published:** 2026-09-23

**Authors:** Haicheng Liu, Huixuan Xu, Chenxuan Rao

**Affiliations:** School of Environmental Science and Engineering, Suzhou University of Science and Technology, Suzhou 215009, China; hui691842@gmail.com (H.X.); szkjdxc@gmail.com (C.R.)

**Keywords:** microplastics, heavy metals, combined pollution, trophic transfer, aquatic food chains, ecological risk

## Abstract

Microplastics (MPs) and heavy metals are widespread co-occurring contaminants in aquatic environments. Their interactions can alter metal mobility and bioavailability and may influence contaminant transfer and toxicity across aquatic food webs. This review critically synthesizes current evidence on MP–metal interactions, trophic transfer, and biological effects across trophic levels. Particular attention is given to the distinction among co-occurrence, bioaccumulation, trophic transfer, and biomagnification. MP–metal adsorption and desorption are jointly regulated by particle properties, metal speciation, and environmental conditions, resulting in substantial variations among exposure systems. Current evidence indicates that MPs can modify metal partitioning, bioaccessibility, and dietary transfer under some conditions. However, persistent particle-bound co-transport and biomagnification across complex aquatic food webs remain insufficiently demonstrated. Biological responses to MP–metal co-exposure are also context dependent, with both strengthened and attenuated effects reported across species and exposure conditions. Stronger or weaker responses under co-exposure should not be interpreted as synergistic or antagonistic without comparison with an appropriate non-interaction reference model. Experimental evidence further suggests that MPs can alter metal bioaccessibility, transport, and biological responses, whereas human evidence remains insufficient to establish increased systemic metal exposure or adverse health outcomes. Future studies should emphasize environmentally relevant exposures, well-defined prey–consumer pathways, quantitative transfer assessment, and standardized approaches for evaluating mixture toxicity.

## 1. Introduction

Plastics are widely used because of their low cost, reliable performance, and ease of processing. According to the United Nations Environment Programme (UNEP), global plastic production exceeded 400 million tons in 2023, almost twice the level reported two decades earlier [1]. Their widespread production and use have also generated large amounts of plastic waste, much of which enters the environment. Microplastics (MPs), commonly defined as plastic particles smaller than 5 mm, have become an emerging contaminant of global concern [2,3,4]. Their small size makes them readily available for ingestion by aquatic organisms. MPs have been detected in a wide range of aquatic taxa, from small invertebrates to large vertebrates [5,6]. Once ingested, MPs can cause physical damage and inflammatory responses, especially when they are retained or accumulated in organisms [7]. MPs can also adsorb other pollutants and may transport them into aquatic food webs [8,9]. These properties make MP pollution an important concern for aquatic ecosystem health.

Metal and metalloid contaminants are persistent in aquatic environments and can accumulate in aquatic organisms. Their potential for trophic transfer and biomagnification varies with contaminant identity, chemical form, species, food-web structure, bioavailability, and exposure conditions [10]. Aquatic organisms can take up metals such as Cd, Pb, Cr, As, Hg, Cu, and Zn through waterborne exposure, sediment resuspension, and ingestion. These metals may accumulate in the gills, liver, kidneys, and muscles of fish, shellfish, and other aquatic animals. Such accumulation can contribute to trophic transfer along food chains [11,12]. Heavy metals can disrupt redox homeostasis, increase the production of reactive oxygen species (ROS), inhibit antioxidant enzymes, and induce lipid peroxidation and DNA damage. These effects can cause cellular dysfunction, histopathological damage, and immune and metabolic disturbances. They may also impair growth and reproduction and affect population stability [13]. In primary producers, heavy metal stress can damage cell membranes, impair photosynthesis and cellular metabolism, and induce oxidative stress. Metal uptake and accumulation by plants and algae can therefore provide an important entry point for metals into aquatic food chains [14]. The increasing occurrence of MPs adds further complexity to heavy metal pollution. MPs can adsorb heavy metals and alter their environmental transport, bioavailability, and tissue distribution. Under co-exposure conditions, MPs may also modify oxidative stress, neurotoxicity, and metal accumulation. Understanding MP–metal interactions and their effects on food-web transfer is important for ecological research and risk assessment [15,16].

Previous reviews have addressed MP–metal pollution from different perspectives, including adsorption and desorption, interfacial mechanisms, combined toxicity, ecological effects, and remediation [17,18,19,20,21,22]. Their scope, ecosystem focus, treatment of trophic transfer, and remaining gaps are compared in Table 1. Despite these advances, a cross-metal synthesis centered on whether and under what conditions MPs modify metal transfer through aquatic prey–consumer links remains limited. Existing reviews either focus mainly on MP–metal interactions and toxicity [17,18,19,21], consider metals as one group within a broader range of MP-associated contaminants [20], or focus on a single metal, such as mercury [22]. In addition, the distinction among co-occurrence, bioaccumulation, trophic transfer, and biomagnification is not always made consistently. Reported increases or decreases in combined toxicity are also sometimes interpreted as synergistic or antagonistic without comparison with an explicit non-interaction reference model.

Accordingly, the main contribution of this review is to critically evaluate whether and under what conditions MPs modify metal uptake and dietary transfer through aquatic prey–consumer links. To address this question, we first examine the adsorption and desorption processes that govern the association of metals with MPs and their subsequent bioavailability. We then evaluate evidence for dietary exposure and trophic transfer across aquatic food chains, with particular attention given to studies using defined prey–consumer pathways. Biological effects of MP–metal co-exposure are compared across trophic levels, and reported mixture effects are interpreted in relation to concentration addition (CA) and independent action (IA). Throughout the review, co-occurrence, bioaccumulation, trophic transfer, and biomagnification are treated as distinct levels of evidence. Potential implications for human dietary exposure are also considered. By linking MP–metal interfacial processes with dietary transfer and biological responses, this review assesses the current evidence for MP-mediated changes in metal transfer and identifies key uncertainties relevant to ecological risk assessment.

## 2. Literature Search and Review Approach

This article was conducted as a structured narrative review. The review focused on the interactions between MPs and metals or metalloids in aquatic environments, with particular attention to adsorption and desorption, combined biological effects, bioaccumulation, dietary exposure, and trophic transfer. A narrative approach was used because the available studies differ substantially in MP characteristics, metal identity and chemical form, exposure design, test organisms, and biological endpoints. These differences limit direct quantitative comparison across studies.

Relevant peer-reviewed literature was identified through searches of the Web of Science Core Collection and Scopus. Search terms were combined according to the topic being examined. Core terms included “microplastic*”, “nanoplastic*”, “metal*”, “metalloid*”, and “heavy metal*”, together with terms related to aquatic environments, biological effects, and trophic transfer. Additional topic-specific terms, such as “adsorption”, “desorption”, “bioaccumulation”, “co-exposure”, “trophic transfer”, and “food web”, were used where appropriate. Specific metal or metalloid names were also included when relevant. The search covered literature available up to September 2026, with no lower publication-year cutoff applied to the narrative review. Relevant studies published before 2020 were identified through the database searches and citation tracking of key primary studies and previous reviews and were retained when they provided important conceptual or experimental evidence relevant to adsorption, trophic transfer, biomagnification, or mixture-toxicity assessment.

Peer-reviewed primary studies were prioritized when they provided experimental or field evidence relevant to the scope of this review. Studies were considered for the main synthesis when they addressed at least one of the following topics: (i) adsorption or desorption of metals by MPs; (ii) biological responses to single or combined MP–metal exposure in aquatic organisms; (iii) changes in metal uptake, bioavailability, or bioaccumulation in the presence of MPs; or (iv) dietary exposure and trophic transfer, with particular attention given to studies using defined prey–consumer pathways. MP-only food-chain studies and metal-only trophic-transfer studies were retained as contextual evidence, but they were distinguished from studies that directly examined MP–metal co-exposure or MP-mediated changes in metal transfer. Studies focused exclusively on terrestrial systems were excluded from the main aquatic evidence synthesis. Methodological and conceptual studies were also included when they were directly relevant to adsorption models, mixture-toxicity assessments, or trophic-transfer metrics. Metal-containing nanoparticles, such as ZnO nanoparticles, were treated as distinct exposure forms rather than as equivalent to dissolved metal species.

Evidence was synthesized qualitatively. Study design features such as comparator treatments, exposure route, prey–consumer relationships, depuration or retention measurements, and quantitative transfer metrics were considered when interpreting the strength and limitations of the available evidence. Co-occurrence, bioaccumulation, trophic transfer, and biomagnification were interpreted as distinct levels of evidence. Simulated gastrointestinal studies were considered when assessing metal desorption and bioaccessibility following ingestion. Human studies were considered only in relation to potential human exposure and health implications.

## 3. Interaction Between Microplastics and Heavy Metals in Aquatic Environments

Interactions between plastic debris and heavy metals were first reported in 2010 [23]. Since then, the adsorption of heavy metals onto MPs has received increasing attention. MPs composed of polyethylene (PE), polypropylene (PP), and polystyrene (PS) can adsorb metals such as Cr, Co, Ni, Cu, Cd, Pb, and Ag [17]. This section examines MP–metal interactions, with emphasis placed on adsorption and desorption behavior and the main factors that control these processes.

### 3.1. Adsorption Behavior and Interaction Mechanisms

Kinetic and equilibrium models are widely used to describe heavy metal adsorption onto MPs [18,24,25]. Kinetic models describe changes in adsorbed amounts over time and can help identify possible rate-limiting processes. The pseudo-first-order (PFO), pseudo-second-order (PSO), and Elovich equations are commonly applied, whereas the Boyd, Weber–Morris, and Bangham models are used to examine film diffusion, intraparticle diffusion, and pore diffusion [18,24]. Equilibrium isotherms describe the relationship between aqueous metal concentrations and the amount adsorbed onto MPs at equilibrium. Commonly used models include the Henry, Langmuir, Freundlich, Temkin, Dubinin–Radushkevich, Redlich–Peterson, and Sips isotherms [18,25].

Across the available evidence, no single kinetic or isotherm model consistently describes all MP–metal systems. A meta-analysis of 3340 data records from 134 publications found that the PSO model and Freundlich isotherm provided the best overall fits, but substantial variations remained among polymers, metals, and experimental conditions [26]. Individual studies illustrate this variability. PSO kinetics described Pb(II) adsorption onto polyvinyl chloride (PVC), PE, and PS well [27], whereas the nonlinear Temkin model provided the best fit for Sr^2+^ adsorption onto polyamide (PA), PS, and PP [28]. Surface conditions can also change the apparent rate-controlling process. For example, intraparticle diffusion dominated trace-metal adsorption onto virgin PS, whereas film diffusion became more important after biofilm colonization [29]. These findings indicate that model performance depends on the polymer type, metal species, surface condition, water matrix, and experimental design.

Model fit, however, should not be interpreted as direct evidence of a specific adsorption mechanism. Similar kinetic or equilibrium behavior can arise from different combinations of mass-transfer and interfacial processes. For example, although Pb(II) adsorption onto PVC, PE, and PS was well described by the PSO model, spectroscopic analysis did not detect new bonds after adsorption, suggesting that a good PSO fit alone does not demonstrate chemisorption [27]. Mechanistic interpretation is therefore stronger when adsorption modeling is combined with independent evidence from surface characterization, spectroscopy, solution chemistry, or molecular simulations [24,25].

Such complementary approaches provide more direct information on metal–MP interactions. For pristine polyamide 6 (PA6) MPs, Fourier-transform infrared spectroscopy and X-ray photoelectron spectroscopy indicated the involvement of amide N and O sites in the binding of Pb(II), Cd(II), and Cu(II), consistent with surface complexation. Reduced Pb(II) and Cd(II) adsorption at higher NaCl concentrations further supported an electrostatic contribution [30]. Molecular simulations have also helped clarify polymer-dependent interactions. For Sr^2+^ adsorption onto PA, PS, and PP, experimental mass-transfer analyses were combined with molecular dynamics simulations, which showed polymer-dependent interaction energies and identified electrostatic interactions as an important attractive contribution, with van der Waals interactions also contributing in some systems [28]. Together, these studies show that mechanistic evidence is strongest when different analytical approaches converge on the same interpretation.

Metal adsorption onto MPs is controlled by multiple interfacial and mass-transfer processes, and their relative importance varies with polymer properties, metal speciation, and experimental conditions [18,26]. Surface characterization, spectroscopy, solution chemistry, and molecular-level analyses can improve mechanistic interpretation. However, the contribution of individual processes often remains uncertain. Differences in experimental conditions add further variability across studies. These limitations help explain the reported heterogeneity and support a closer examination of the factors that control MP–metal interactions.

### 3.2. Factors Affecting Heavy Metal Adsorption onto Microplastics

#### 3.2.1. Microplastic Properties

Polymer composition and surface properties are important determinants of metal adsorption onto MPs. Common polymers in aquatic environments include PE, PS, PP, polyethylene terephthalate (PET), PVC, and PA [31]. These polymers differ in specific surface area, polarity, crystallinity, surface charge, and functional groups. A larger specific surface area usually provides more available binding sites, although surface area alone does not determine adsorption capacity. For Cd(II), adsorption capacities increased from 113.5 mg kg^−1^ for PE to 151.4 mg kg^−1^ for PVC, following the same order as their specific surface areas [32]. In contrast, PP showed a higher Sr^2+^ adsorption capacity (52.4 μg g^−1^) than PS (51.4 μg g^−1^) and PA (31.8 μg g^−1^), despite having the smallest specific surface area [28]. Surface chemistry also affects metal binding. Chlorine-containing polar groups on PVC and increased surface polarity after PE chlorination have been associated with greater adsorption of Pb, Cu, and Cd [32,33].

Particle size affects adsorption partly through changes in surface area and the number of exposed binding sites. Smaller particles often show greater adsorption capacity. For polytetrafluoroethylene (PTFE), the Langmuir-fitted maximum As(III) adsorption capacity decreased from 1.05 to 0.91 mg g^−1^ as particle size increased from 100 to 1000 nm to >10,000 nm [34]. Similar size-dependent decreases in the adsorption of Cr(III) and Pb(II) were reported for PE, PP, and PET MPs [35]. The relationship between particle size and adsorption is not always linear. Very small particles may agglomerate, which can reduce the effective specific surface area and the number of accessible adsorption sites.

Aging alters both the surface morphology and chemistry of MPs. Physical and chemical weathering can increase surface roughness, form pores and cracks, and introduce oxygen-containing functional groups [36,37,38]. After 12 months of ultraviolet aging in seawater, aged PE, PP, and PET adsorbed 1.45–2.92 times more Cu(II) than the corresponding unaged particles [39]. Natural weathering can cause more complex surface changes. Weathered PE-rich particles collected from the Xiangjiang River contained a porous organic surface layer and adsorbed more Pb(II) than pristine PE. Disruption of this layer reduced adsorption, indicating that surface-associated organic matter contributed to Pb(II) binding [40]. Mechanical weathering of low-density polyethylene (LDPE) and PET also altered Pb(II) and Zn(II) uptake, but the response depended on the polymer, metal, and weathering medium [41]. Aging therefore does not produce a uniform increase in adsorption across MP–metal systems.

Biofilm colonization is another important form of MP surface modification in aquatic environments. Microorganisms and extracellular polymeric substances (EPS) can change surface roughness, hydrophilicity, surface charge, and functional-group composition. Carboxyl, amino, and phenolic hydroxyl groups in biofilms and EPS can provide additional binding sites for metals [29,42]. In one study, the Langmuir-derived maximum Cu(II) adsorption capacity increased from 643.1 μg g^−1^ for virgin PS to 1048.0 and 819.9 μg g^−1^ after biofilm colonization in two aquatic environments [29].

Biofilm formation also shifted the dominant uptake process from intraparticle diffusion to film diffusion. Fungal colonization of PS increased surface functional groups and enhanced Cu adsorption and Cr(VI) reduction [42]. Microbial colonization and biodegradation of poly(butylene succinate) (PBS) also increased Pb(II) adsorption [43]. The effect of biofilm colonization varies with polymer properties, particle size, biofilm characteristics, and environmental conditions.

#### 3.2.2. Environmental Conditions

Environmental conditions, including pH, salinity, and natural organic matter (NOM), can influence metal adsorption onto MPs. pH affects both MP surface charge and metal speciation. As pH increases, surface functional groups generally become more deprotonated, which makes MP surfaces more negatively charged. This change can promote the adsorption of cationic metals such as Cd(II), Pb(II), Cu(II), and Fe(III) through electrostatic attraction. At a higher pH, metal hydrolysis or hydroxide precipitation may also occur. These processes can reduce dissolved metal concentrations and affect the apparent adsorption behavior. The influence of pH therefore depends on both MP surface chemistry and the aqueous form of the target metal [44,45].

Salinity and ionic composition can also affect metal adsorption. In 0.01 mol L^−1^ NaCl, the maximum Pb(II) adsorption capacities of PVC, PS, and PE MPs were 476.9, 246.4, and 531.6 μg g^−1^, respectively. When the NaCl concentration increased to 0.1 mol L^−1^, the corresponding values decreased to 328.0, 227.1, and 459.0 μg g^−1^ [27]. Higher ionic strength can weaken electrostatic attraction by screening surface charges and compressing the electrical double layer. Coexisting ions may also compete with target metals for binding sites [44]. Particle aggregation under high-ionic-strength conditions may further reduce the surface area available for adsorption.

The effect of NOM depends on its concentration, composition, and interaction with MP surfaces. Humic acid (HA), a common component of NOM, is usually negatively charged and contains functional groups such as carboxyl, hydroxyl, and phenolic hydroxyl groups. Dissolved HA can bind metal ions through electrostatic interactions and complexation. This binding can reduce the fraction of metals available for adsorption onto MPs, allowing HA to compete with MPs for some metal ions [46,47]. HA can also adsorb onto MP surfaces and form an organic coating. Functional groups in this coating may provide additional coordination sites for metals. The coating can also alter the zeta potential and electrostatic properties of MP surfaces, which may increase the adsorption of some metal ions [48]. The net effect of NOM depends on its concentration and composition, as well as MP surface properties and solution chemistry.

#### 3.2.3. Heavy Metal Properties and Speciation

Heavy metal ions differ in ionic radius, hydration energy, charge density, and coordination behavior. These properties can affect their interactions with MP surfaces and influence adsorption capacity, adsorption rate, and binding behavior. MPs often show relatively high affinity for Pb(II). This has been related to the physicochemical properties of Pb(II), including its ionic radius and tendency to form surface complexes. Cd(II) may show lower adsorption because of weaker interactions with some MP surfaces [49]. Yang et al. [50] reported a similar pattern for 150 μm polyacrylate (PAT) MPs. The maximum adsorption capacities followed the order Pb(II) (34.68 mg/g) > Cu(II) (29.85 mg/g) > Cd(II) (12.31 mg/g). This difference shows that adsorption cannot be explained by MP properties alone.

The metal oxidation state and aqueous speciation can also affect adsorption onto MPs. Cr(VI), for example, mainly occurs in aquatic systems as anionic species such as HCrO_4_^−^ and CrO_4_^2−^. Its adsorption varies with MP surface charge, aging state, pH, and the presence of coexisting ions. Ultraviolet aging can increase surface roughness and introduce oxygen-containing functional groups, which may alter Cr(VI) adsorption. Under the tested conditions, aged MPs showed greater Cr(VI) adsorption than pristine particles. Aged PS MPs may also promote the reduction of Cr(VI) to Cr(III). These findings suggest that MPs can affect not only metal adsorption and transport but also metal speciation and environmental fate [51,52].

#### 3.2.4. Integrated Effects of Controlling Factors

Metal adsorption onto MPs is jointly influenced by particle properties, metal characteristics, and environmental conditions. A meta-analysis of 3340 data records from 134 publications identified metal concentration, MP concentration, specific surface area, and pH as major predictors of adsorption [26]. Experimental conditions showed the strongest overall influence in the compiled dataset. These results describe broad patterns rather than a fixed hierarchy of controlling factors. Their relative importance varies among polymers, metals, water matrices, and experimental designs.

The effects of individual factors are also interdependent. Polymer identity alone cannot predict adsorption because particle size, surface chemistry, aging, and biofilm colonization can alter the number and accessibility of binding sites [28,30,49,50]. Aging is a clear example. Its effect depends on changes in surface oxidation, organic coatings, mineral deposits, and the surrounding water chemistry [40,41,53,54]. Metal properties and aqueous speciation can further modify these surface-dependent interactions [29,42,43].

Water chemistry can further modify these surface-mediated processes. The meta-analysis found a nonlinear relationship between pH and adsorption, with the highest adsorption occurring at approximately pH 5.0–6.1 [26]. Ionic strength and dissolved organic matter (DOM) showed no significant overall effects in the compiled dataset. These results should be interpreted cautiously because relatively few studies examined these variables. The absence of a significant overall effect does not exclude important responses in specific MP–metal systems.

Cross-study comparisons are further affected by differences in exposure conditions and data treatment. Metal and MP concentrations, particle size, surface area, surface condition, pH, ionic composition, organic matter, and water matrix should be considered when comparing adsorption results. Adsorption values normalized to particle mass are not directly equivalent to those normalized to surface area. Measured equilibrium adsorption should also be distinguished from maximum capacities estimated from isotherm models because fitted values depend on the tested concentration range and model selection [17,21,26].

### 3.3. Desorption and Metal Release Under Environmental and Simulated Gastrointestinal Conditions

The interpretation of desorption and metal release depends on the source and binding form of the metal. Desorption refers to the release of previously adsorbed metals from MP surfaces after a change in interfacial equilibrium [17,21]. In contrast, metals released from field-collected or weathered plastics may originate from surface contamination, additives, pigments, or associated mineral phases [55,56]. Total metal release should therefore not be interpreted as desorption unless the previously adsorbed fraction is identified. A metal mass balance can further improve source attribution.

Changes in water chemistry can alter the stability of MP-bound metals. In one study, pristine and weathered LDPE were first loaded with Pb(II) and Zn(II) at pH 7.0 and then transferred to metal-free water at pH 5.0. After 120 h, Pb release was slightly higher from pristine LDPE, whereas Zn release increased from 10.8% for pristine LDPE to 71.9% for weathered LDPE [43]. The opposite responses of Pb and Zn show that aging does not affect metal retention in a uniform manner. Metal identity, surface condition, and the receiving medium all influence desorption.

Simulated gastric conditions produced substantial Cr(VI) desorption from PA and polylactic acid (PLA) MPs. The tested materials included pristine and ultraviolet (UV)-aged PA and PLA MPs. After 36 h at pH 2.0, Cr(VI) desorption was approximately 67% from pristine PA and 43% from aged PA. The corresponding values for pristine and aged PLA were approximately 63% and 35%, respectively [51]. Cu showed a different pattern on PS. Under simulated gastric conditions, Cu was completely released from ozone-aged PS, while the release from solar-aged and lake-aged PS was 72% and 58%, respectively [54]. Little Cu was released from virgin PS because little had been adsorbed during the loading stage. Adsorption capacity alone is therefore not sufficient to predict metal release during digestion.

Evidence from field-collected plastics requires additional caution. Weathered PE-rich particles released several metals under simulated gastric conditions, with reported release fractions ranging from 14.68% for Pb to 97.80% for Cr [55]. The particles contained metals in several operationally defined fractions, and their exact sources could not be resolved. Artificial cutting of naturally aged macroplastics also limits direct comparison with MPs formed by environmental fragmentation. Similar uncertainty applies to studies using plastics that contain metal-bearing pigments or additives.

Particle-size reduction can also alter metal release, but the response is metal specific. A simulated rainbow trout digestion model compared LDPE pellets (3 × 3 mm) with cryogenically milled LDPE MPs (modal diameter = 272 μm; 10 vol% < 100 μm). Gastric Pb release increased from 0.5 ± 0.2 to 24.5 ± 0.3 μg L^−1^ after milling, while Cr release decreased from 163 ± 1 to 131 ± 2 μg L^−1^ [56]. The LDPE material contained pigments; therefore, part of the measured metal may have originated from the plastic itself rather than from environmental adsorption. This source uncertainty should be considered when interpreting metal release from weathered or manufactured plastics.

Current evidence indicates that MP–metal interactions are strongly context dependent. Particle properties, surface aging, metal identity and speciation, and water chemistry can alter adsorption, desorption, mobility, and potential bioaccessibility [17,21,26]. Whether these physicochemical changes consistently increase biological uptake or food-web transfer remains unresolved. Interpretation is constrained by heterogeneous experimental conditions and by incomplete characterization of metal speciation, dissolved and particle-associated fractions, and mass balance. Physicochemical studies therefore support mechanistic plausibility, but they do not by themselves demonstrate trophic transfer or biomagnification.

## 4. Trophic Transfer of Microplastics and Heavy Metals in Aquatic Food Chains

MPs and metals can co-occur in water, sediments, and aquatic organisms after entering aquatic ecosystems. Both pollutants may also enter food webs through feeding. Co-occurrence means that MPs and metals are present in the same environmental matrix or organism, but it does not by itself demonstrate physical association, interaction, or co-transport [57]. Trophic transfer refers to the movement of a contaminant from prey to consumer through feeding [58]. Bioaccumulation refers to the uptake and retention of a contaminant within an organism through all relevant exposure routes. Biomagnification occurs when dietary uptake results in a higher contaminant concentration in the consumer than in its prey. A biomagnification factor (BMF) quantifies this change within a defined prey–consumer relationship. A trophic magnification factor (TMF) describes the average change in contaminant concentration with increasing trophic position across a food web [59]. Figure 1 summarizes the potential pathways by which MPs and metals enter aquatic organisms, move through trophic levels, and contribute to potential human dietary exposure.

### 4.1. Trophic Transfer of Microplastics

Controlled prey–consumer experiments have shown that MPs can move between trophic levels in both freshwater and marine food chains [60]. Early work showed that 0.5-μm PS particles ingested by mussels could subsequently be detected in the hemolymph and tissues of the shore crab *Carcinus maenas* [61]. Later experiments confirmed similar transfer across a range of aquatic prey–consumer systems. These studies establish feeding as a route of MP transfer, but they differ markedly in particle size, exposure concentration, food-chain length, and the duration of consumer exposure.

Dietary exposure can increase short-term MP uptake compared with direct waterborne exposure. In a *Favella* spp.–*Menidia beryllina* model, dietary exposure produced greater particle uptake than direct exposure, although most particles were eliminated within 24 h [62]. A similar pattern was observed in Baltic Sea littoral food chains. *Praunus flexuosus* ingested substantially more MPs through contaminated prey than through waterborne exposure, and transfer continued to the higher-level consumer *Palaemon elegans* [63]. Particle abundance decreased along the food chain, and the experiment did not include a depuration period. The results therefore demonstrate trophic transfer but do not support biomagnification or persistent accumulation.

Retention after dietary transfer also varies among experimental systems. PS particles transferred from *Bellamya aeruginosa* to *Mylopharyngodon piceus* were detected mainly in the gastrointestinal tract, although smaller amounts were also found in muscle [60]. In an *Artemia salina*–*Litopenaeus vannamei*–*Oreochromis niloticus* food chain, MPs remained mainly in the gut, intestinal epithelium, and feces, and all reported that bioaccumulation-related factors were below 1 [64]. Experiments with *Daphnia magna* and *Danio rerio* further showed that dietary exposure produced greater uptake than waterborne exposure, but 87.86–98.22% of the detected particles were recovered in feces after 12 h when prey were supplied [65]. These results indicate that trophic transfer can increase exposure without necessarily producing long-term retention in consumers.

Field studies provide greater environmental relevance but weaker evidence for direct prey-to-consumer transfer. In the Zhoushan fishing ground, MP abundance was positively correlated with the trophic level across fish, crustaceans, zooplankton, and zoobenthos (*r* = 0.717, *p* < 0.05) [66]. The study identified plausible feeding pathways, but individual particles were not tracked from prey to consumers. Uptake from water or sediments could not be excluded, and differences in habitat, feeding behavior, body size, and particle retention may also have contributed to the observed pattern. Such field associations are therefore consistent with food-web transfer but do not by themselves demonstrate trophic transfer or biomagnification.

Current evidence supports the transfer of MPs through defined prey–consumer links, particularly under controlled feeding conditions. The strength of evidence is lower for persistent retention and progressive accumulation across trophic levels. Dietary exposure can increase short-term particle uptake, whereas rapid elimination has been reported in several experimental systems [63,64]. Biomagnification should therefore not be inferred from the detection of MPs at higher trophic levels alone. Evidence from complex natural food webs remains limited, and stronger conclusions require matched prey–consumer measurements together with information on uptake, depuration, and particle retention [20].

### 4.2. Trophic Transfer of Microplastics and Heavy Metals

MPs may alter metal transfer through aquatic food chains by changing metal partitioning within prey and metal bioaccessibility during digestion. After contaminated prey are consumed, metals may remain associated with MPs, desorb into digestive fluids, or be released from prey tissues and taken up independently of the particles. MP retention and metal elimination can further influence metal exposure in consumers [18,21]. These pathways are mechanistically plausible, but they represent different forms of evidence and should not be treated as equivalent.

Field studies mainly show co-occurrence rather than transfer through defined feeding links. MPs and multiple metals have been detected together in oysters, and scanning electron microscopy coupled with energy-dispersive X-ray spectroscopy (SEM–EDS) identified several metals on some recovered particles [67]. In freshwater fish, MP abundance was also positively associated with a metal pollution index [68]. Such observations show that MPs and metals can occur in the same organisms. They do not establish that MPs caused metal accumulation or that the metals were transferred with the particles through feeding.

Controlled sequential-feeding experiments provide more direct evidence of transfer through defined trophic links. In a *Paramecium caudatum*–*Danio rerio* model, ciliates were exposed to PS MPs and Cd(II), washed, and then fed to zebrafish. Both MPs and Cd were subsequently detected in the consumers [69]. Washing reduced the contribution of direct waterborne exposure and strengthened the evidence for dietary transfer. The study did not track whether Cd remained associated with MPs during ingestion or digestion. A separate experiment exposed juvenile *Hippocampus kuda* to high-density polyethylene (HDPE) MPs preloaded with Cu, Cd, and Pb and mixed with thawed mysids [70]. MPs and metals accumulated during the exposure period, but the absence of a metal-only dietary treatment prevented the specific contribution of MPs from being quantified. These studies demonstrate dietary co-exposure, but not particle-bound co-transport.

Stronger evidence that MPs can modify dietary metal transfer comes from a *Chlorella vulgaris*–*Daphnia magna* model [71]. Algae were exposed to ZnO nanoparticles alone or together with PS MPs, and free particles were removed before feeding. Although total Zn in the prey was lower in the combined treatment than in the ZnO-only treatment, Zn concentrations in *D. magna* after 21 days were higher (3.10 ± 0.42 vs. 1.61 ± 0.10 mg g^−1^ dry weight). The reported BMF for total Zn increased from <0.90 in the ZnO-only treatment to 2.11 after 21 days in the presence of PS MPs. The estimated Zn uptake-rate constant also increased by 103.0%, whereas the depuration-rate constant changed little. These results indicate that prior MP exposure of the prey increased dietary Zn transfer and retention under the tested conditions. ZnO nanoparticles should be considered a distinct metal-containing particle form rather than equivalent to dissolved Zn^2+^.

Follow-up experiments with the same prey–consumer model helped explain this effect [72]. PS MPs damaged algal cells and increased the release of prey-associated Zn during simulated digestion. Intestinal Zn in *D. magna* was 1.70 times higher than in the ZnO-only treatment. Because free PS MPs were removed before feeding, the increased Zn transfer cannot be attributed to direct transport of MP-bound Zn into the consumer. The evidence instead supports an indirect mechanism involving changes in prey properties and greater digestive bioaccessibility of Zn. The separate contributions of particulate ZnO and dissolved Zn^2+^ could not be resolved.

The available evidence supports several clear distinctions. Co-occurrence of MPs and metals in field organisms does not demonstrate trophic transfer. Detection of both pollutants after controlled feeding provides stronger evidence for a shared dietary pathway, but it does not establish persistent particle-bound transport. Comparisons of metal uptake or retention between treatments with and without MPs provide stronger evidence for MP-mediated changes in dietary metal transfer [71,72]. Evidence for persistent particle-bound co-transport and biomagnification remains limited. This limitation reflects the small number of defined feeding studies, incomplete comparator treatments, limited depuration and retention measurements, and the scarcity of metal-speciation, mass-balance, and quantitative transfer data. Biomagnification should therefore be interpreted within the specific metal form, prey–consumer system, exposure route, and study duration rather than generalized across aquatic food webs.

## 5. Effects of Microplastics and Heavy Metals on Different Trophic Levels

Changes in metal partitioning and bioaccessibility can alter both the amount and chemical form of metal available to organisms, thereby changing the relative contributions of waterborne and dietary exposure. Co-exposure may also modify biological responses through processes that occur after uptake. Most available studies do not clearly distinguish these exposure-mediated effects from post-uptake interactions [18,21]. CA and IA provide established reference models for evaluating whether mixture effects deviate from additivity [73,74].

CA treats the mixture components as dilutions of one another and is commonly applied to substances with similar modes of action. For a mixture of n components that produces an x% effect, CA is expressed as:

(1) $$\sum_{i=1}^{n}\frac{{c^{*}}_{i}}{{ECx}_{i}}\;=\;1$$

where ${c^{*}}_{i}$ is the concentration of component i in a mixture producing an x% effect, and ${ECx}_{i}$ is the concentration of the same component producing an x% effect when tested individually. Each ratio ${c^{*}}_{i}/{ECx}_{i}$ represents a toxic unit, and a sum of one indicates conformity with the CA prediction. For a mixture with a specified composition, the predicted effect level is obtained by identifying the value of x that satisfies Equation (1) [73,75].

IA assumes that the mixture components act independently on different biological subsystems. For a response parameter that decreases with increasing exposure concentration, such as survival, the expected mixture response is calculated as:

(2a) $$E\left(c_{mix}\right)\;=\;E\left(c_{1}+\cdots +c_{n}\right)\;=\;\prod_{i=1}^{n}E(c_{i})$$

For a response parameter that increases with exposure concentration, such as mortality or inhibition, the expected mixture effect is calculated as:

(2b) $$E\left(c_{mix}\right)\;=\;E\left(c_{1}+\cdots +c_{n}\right)\;=\;1-\prod_{i=1}^{n}[1-E\left(c_{i}\right)]$$

where $E(c_{i})$ is the response produced by component i when tested individually at the concentration at which it is present in the mixture, and $E(c_{mix})$ is the IA-predicted mixture response, with response values scaled from 0 to 1 [73,75,76].

The interpretation of mixture effects depends on the selected reference model and experimental context. Both CA and IA require concentration–response relationships for the individual components under comparable conditions. These models provide operational no-interaction references rather than direct evidence of the underlying toxicological mechanisms. Their assumptions may only be approximately satisfied in complex environmental mixtures, and CA and IA can produce similar predictions at low effect levels. The magnitude and direction of deviations may also vary with the mixture ratio and exposure level [74,76]. Agreement with the selected model is consistent with additivity, whereas a greater or weaker effect than predicted is consistent with synergism or antagonism, respectively. A combined response that is stronger or weaker than either single-stressor response does not, by itself, demonstrate either interaction type. Classification requires a reproducible and statistically supported deviation from the selected reference model, together with consideration of uncertainty and biological relevance [76,77].

Organism-level co-exposure studies and trophic-transfer experiments provide different types of evidence. Table 2 summarizes studies comparing MP-only, metal-only, and combined exposures within individual aquatic species and the biological responses observed across trophic groups. Table 3 focuses on defined prey–consumer pathways and summarizes the transfer and accumulation of MPs and metals together with biological responses in consumers.

### 5.1. First Trophic Level: Primary Producers

Primary producers form the base of aquatic food webs and include phytoplankton, macroalgae, and aquatic vascular plants. Exposure to MPs, metals, and their mixtures can affect growth, photosynthetic performance, pigment content, redox balance, and metal accumulation. The direction and magnitude of these responses vary among producer groups and exposure conditions [85,86].

Single-pollutant studies provide useful context for this variability. Different polymers can produce contrasting responses even within closely related producer systems. PE, PET, and PVC promoted the growth of *Chlorella* sp. L38 under some conditions, whereas PP caused transient inhibition; all four polymers inhibited *Phaeodactylum tricornutum* [87]. Metal exposure also produces species- and endpoint-dependent responses [88]. These findings show that changes in growth alone do not fully describe the physiological effects of either MPs or metals.

Direct MP–metal co-exposure can strengthen or attenuate biological responses, but the direction is not consistent. In *Microcystis aeruginosa*, PE–Cd exposure caused greater chlorophyll inhibition and higher microcystin content than Cd alone [89]. The study included matched single-exposure treatments, allowing direct comparison at the tested concentrations. However, only one concentration of each pollutant was used, and Cd partitioning among dissolved, particle-associated, and cell-associated fractions was not measured. The results therefore show stronger biological effects under the tested conditions, but they do not demonstrate increased Cd bioavailability or formal synergism.

Polymer identity can further alter the response within the same organism–metal system. In *Chlorella vulgaris*, PE enhanced membrane damage and apoptosis at some Zn(II) concentrations, whereas PVC attenuated several Zn-related growth and oxidative responses [90]. PVC also showed greater Zn sorption than PE. This comparison supports a role for polymer-dependent sorption and particle–cell interactions. The tested combinations did not provide a complete concentration–response design, and the mixture effects were not evaluated against CA or IA. The responses are therefore better described as polymer- and concentration-dependent changes rather than synergistic or antagonistic effects.

Evidence for altered metal exposure is stronger when bioavailability or internal metal levels are measured together with biological responses. PS reduced Pb bioavailability and growth inhibition in *Chlamydomonas reinhardtii*, with a greater reduction when Pb was preloaded onto the particles before exposure [91]. Aged PS and PVC also reduced Cu- and Cd-related growth inhibition in *C. vulgaris*, although a complete metal mass balance was not available [92]. In submerged macrophytes, PE reduced dissolved Cu and acute Cu toxicity, whereas Cu-loaded PE caused toxicity after transfer to a low-Cu medium [93]. These studies indicate that MPs can reduce local metal availability through sorption while still retaining the capacity to transport and release metals under changing conditions.

Changes in biological effects do not always reflect changes in metal uptake. In *Vallisneria natans*, PVC reduced biomass in the presence and absence of Cd but did not significantly change plant Cd concentrations [94]. The additional growth inhibition was therefore more consistent with particle-related stress than with increased Cd accumulation. PS also modified Pb-related responses in *Potamogeton crispus* only at selected exposure levels and endpoints, and similar effects were not consistently observed in *Vallisneria denseserrulata* [95]. Such variations may reflect not only species-specific sensitivity but also differences in exposure pathways and environmental compartments. Results from suspended microalgal systems therefore should not be directly extrapolated to rooted macrophytes.

The available evidence does not support a consistent increase or decrease in metal toxicity caused by MPs in primary producers. Stronger responses occur in some systems, whereas sorption or aggregation can reduce the bioavailable metal fraction and attenuate toxicity [89,91,93]. In other systems, MPs alter physiological responses without measurable changes in metal accumulation, showing that changes in toxicity cannot be assumed to reflect changes in metal uptake [94].

### 5.2. Second Trophic Level: Primary Consumers

Primary consumers connect basal production with higher trophic levels and include zooplankton, filter-feeding bivalves, and herbivorous or detritivorous benthic invertebrates. They can encounter MPs and metals directly through water or sediment and indirectly through contaminated food. Feeding conditions and particle properties can influence MP ingestion and retention [96,97,98,99]. These differences also contribute to variations in co-exposure responses.

Direct MP–metal co-exposure does not produce a consistent biological response. In *Daphnia magna*, the response to Ni differed between plain and carboxylated PS, indicating that surface functionalization can modify mixture effects [100]. The observed pattern was based on acute immobilization and cannot be extended directly to chronic exposure. Exposure duration can also change the apparent response. PVC reduced the acute toxicity of Cd in *D. magna*, as indicated by an increase in the 48 h median effective concentration (EC_50_) from 215.3 to 343.4 μg L^−1^, whereas longer co-exposure impaired development, reproduction, locomotion, intestinal structure, and energy metabolism [81]. Acute attenuation therefore does not imply reduced effects during prolonged exposure.

Biological endpoints alone do not show whether MPs have altered metal exposure. Stronger evidence is obtained when the internal metal burden is measured together with biological responses. MP co-exposure increased Cd accumulation in *D. magna* by 74.35% and was accompanied by greater reproductive impairment [101]. Greater Hg accumulation and stronger Hg-related effects were also reported in the marine copepod *Tigriopus japonicus* during multigenerational exposure [102]. The opposite pattern has also been observed. MPs reduced Hg bioconcentration in the freshwater bivalve *Corbicula fluminea*, although filtration activity declined and lipid peroxidation increased [103]. In *Centropages velificatus*, PET–Ni co-exposure produced reproductive responses similar to those caused by Ni alone [104]. MP-mediated changes in metal accumulation therefore vary with polymer properties, metal identities, species, exposure durations, and endpoints.

Feeding studies provide additional context for the role of exposure route. MP-only experiments show that particles can be transferred from contaminated producers to primary consumers, but the resulting biological effects vary among systems [105,106]. Metal-only studies also show that dietary exposure can produce different tissue distributions and biological effects from dissolved exposure. In *Acartia hudsonica* and *A. tonsa*, ingestion of metal-contaminated phytoplankton increased internal Hg or Cd burdens and reduced reproductive performance, whereas dissolved exposure resulted mainly in metal deposition on the exoskeleton [107]. These studies show that dietary exposure can modify contaminant delivery, but they do not by themselves demonstrate an interaction between MPs and metals.

Direct evidence for combined MP–metal effects through defined producer–consumer pathways remains limited (Table 3). In the *Chlorella vulgaris–Daphnia magna* system, prior exposure of algae to PS MPs and ZnO nanoparticles increased Zn accumulation and adverse effects in the consumer [71]. Follow-up experiments linked this response to greater damage to algal cells and an increased release of prey-associated Zn during digestion [72]. Because PS MPs were removed before feeding, these findings support an indirect trophic mechanism in which prior MP exposure alters prey properties and increases the digestive bioaccessibility of Zn rather than direct co-transport of MP-bound metal.

Current evidence does not indicate a consistent enhancement or attenuation of metal toxicity by MPs in primary consumers. Direct co-exposure responses vary with MP surface properties, exposure durations, metal identities, species, and biological endpoints [81,100,102,103]. Evidence is stronger when changes in metal accumulation or bioaccessibility are measured together with biological responses [71,72,101]. Defined producer–consumer studies also indicate that MPs can modify consumer exposure indirectly by altering contaminated prey, but evidence for this pathway remains limited to a small number of simplified systems.

### 5.3. Higher Trophic Levels: Secondary and Higher Consumers

Secondary and higher consumers include predatory and omnivorous fish, crustaceans, and other aquatic animals. They can acquire MPs and metals directly from the surrounding environment or through contaminated food and prey. The relative contribution of these exposure routes varies among species and experimental conditions. Direct co-exposure should therefore be distinguished from dietary and prey-mediated exposure when interpreting changes in metal accumulation and toxicity [20,58].

Direct co-exposure studies show that MP effects on metal accumulation can change with exposure conditions. In zebrafish, 5 μm PS increased Cd accumulation and toxicity at 15 μg L^−1^ Cd but reduced both responses at 150 μg L^−1^ [108]. At the higher Cd concentration, PS also reduced the freely available Cd fraction in the exposure water. This reversal within the same organism–polymer–metal system shows that MPs do not act as fixed carriers of metals. Their effects depend on the metal concentration and partitioning.

Polymer type can also alter internal metal exposure. In zebrafish, intestinal Cd concentrations increased from 5.84 μg g^−1^ with Cd alone to 8.49 and 12.36 μg g^−1^ with PVC–Cd and PP–Cd, respectively [109]. Both mixtures caused greater intestinal injury, inflammation, and oxidative responses than Cd alone. The effect was stronger with PP. These findings support polymer-dependent changes in Cd accumulation and toxicity. Because the mixtures were not evaluated against CA or IA predictions, the responses should not be classified as synergistic.

Changes in metal burden do not always occur in the same direction. In *Channa punctatus*, PVC–Cu co-exposure increased intestinal Cu accumulation and produced stronger oxidative and intestinal injury than Cu alone [83]. In contrast, PVC reduced intestinal Pb concentrations in *Cyprinus carpio*, together with lower lipid peroxidation and inflammatory responses [110]. Feeding activity also declined during co-exposure, suggesting that a reduced Pb intake contributed to the lower internal burden. These contrasting findings show that MP effects on metal toxicity can result from changes in partitioning, uptake, feeding behavior, or retention.

Dietary co-exposure represents a different exposure context. Juvenile *Litopenaeus vannamei* fed PE and Cd together showed a lower final body weight and specific growth rate than animals exposed to either pollutant alone [82]. Because both pollutants were added directly to formulated feed, this experiment demonstrates dietary co-exposure rather than trophic transfer through a contaminated prey–consumer link. MP-only and metal-only feeding studies also show that dietary exposure can produce biological effects even when retention or transfer is limited [60,64,111]. These studies show that trophic exposure can produce biological effects even when transfer or accumulation is limited, but they do not establish that MPs modify metal toxicity.

Evidence directly linking combined exposure to prey-mediated toxicity in higher consumers remains scarce (Table 3). In a *Paramecium caudatum–Danio rerio* model, ciliates were exposed to Cd alone or to PS MPs and Cd, washed, and then fed to zebrafish [69]. The combined treatment caused greater changes in liver development, metabolism, gut microbiota, inflammation, DNA damage, and apoptosis than Cd alone. Washing reduced residual waterborne exposure and strengthened the evidence for prey-mediated co-exposure. The absence of an MP-only trophic treatment and a formal mixture reference model prevents these responses from being classified as synergistic.

A 90-day study using the same feeding link reported effects on heart, liver, and gonadal development [84]. Repeated washing of contaminated prey again reduced the contribution of residual waterborne exposure. Developmental and endocrine pathways were altered, but only the control and combined-exposure groups were included. The individual contributions of MPs and Cd could therefore not be separated.

These prey–consumer studies demonstrate that MPs and metals can reach higher consumers through contaminated prey and produce biological effects [69,84]. They provide weaker evidence that MPs increase metal transfer across the trophic link. Neither study quantified transfer efficiency or reported a BMF based on matched prey and consumer metal burdens. The form of Cd during transfer also remained unresolved. Without measurements that distinguish MP-bound Cd from other prey-associated Cd, particle-bound co-transport cannot be confirmed.

Current evidence in higher consumers therefore supports the toxicological relevance of direct and prey-mediated MP–metal co-exposure. Changes in internal metal burden depend on the metal concentration, polymer type, feeding behavior, and exposure route [83,109]. Formulated-feed studies demonstrate dietary co-exposure but not trophic transfer [82]. Defined prey–consumer studies show biological effects after prey-mediated exposure, but evidence remains insufficient for consistent MP-mediated enhancement of metal transfer, persistent particle-bound co-transport, or biomagnification across higher trophic levels.

### 5.4. Potential Human Exposure and Health Implications

Humans can be exposed to MPs and metals through food, drinking water, inhalation, and potentially dermal contact [112,113,114]. In the context of aquatic food webs, dietary exposure is the most relevant pathway. MPs have been detected in commercially consumed fish, crustaceans, and mollusks, although reported abundances vary widely among species, regions, and analytical methods [115]. MPs have also been detected in edible tissues, supporting the possibility of dietary intake from aquatic foods [116]. Their occurrence in food, however, does not demonstrate trophic transfer to humans or a toxicologically relevant internal dose.

For MP–metal co-exposure, the key question is whether co-ingestion changes metal release, bioaccessibility, intestinal transport, or subsequent biological responses. Gastrointestinal models provide direct mechanistic evidence for some of these processes. In a simulated digestion and intestinal epithelium model, ultraviolet-aged PET micro- and nanoplastics (MNPs) increased the epithelial translocation of As and Pb and enhanced intracellular oxidative stress during co-exposure [117]. Although the combined treatment produced a stronger oxidative response, formal synergism cannot be established because the mixture was not evaluated against a CA or IA reference model. These findings support altered contaminant transport and cellular responses under the tested in vitro conditions, but their relevance to systemic human toxicity remains uncertain.

Modeling and digestion studies also show that metal release from MPs does not directly represent systemic exposure. A scenario-based assessment predicted greater gastrointestinal exposure to Cr(VI) and Pb(II) from aged MPs than from virgin MPs, with marked differences between gastric and intestinal phases [118]. No immediate health risk was identified under the modeled conditions. A separate in vitro digestion study showed that risk estimates based on bioaccessible metal fractions were higher than those based on simulated bioavailable fractions [119]. These findings indicate that gastrointestinal release does not necessarily correspond to the fraction potentially available for intestinal uptake.

Human evidence remains limited. MPs have been detected in multiple human organs and biological samples, including stool, meconium, semen, and urine [120,121]. In a prospective multicenter observational study, MNPs were detected in carotid plaques, and their presence was associated with a higher incidence of the composite outcome of myocardial infarction, stroke, or all-cause death during a mean follow-up of 33.7 months [122]. Evidence specific to MP–metal co-exposure comes from a cross-sectional study of 68 preschool children. MPs and Cd were detected in all fecal samples, and their concentrations were inversely associated. MP–Cd co-exposure was also associated with differences in gut microbiota composition and predicted metabolic functions [123].

Assessment of the human health implications of MP–metal co-exposure remains limited by incomplete evidence linking external exposure, internal dose, and health outcomes. Human exposure estimates are difficult to compare because they depend on the exposure routes and matrices considered, as well as the particle-size range and analytical methods used [112]. Human biomonitoring is further complicated by differences in sample preparation and analytical methods, together with the risk of procedural contamination, which can affect data interpretation and comparability among studies [120,121,124]. These limitations are particularly important for MP–metal co-exposure because the detection of MPs and metals in the same biological sample does not by itself demonstrate that MPs increase metal uptake or systemic metal burden. These observational findings do not establish that MP-mediated changes in metal exposure are responsible for the observed health-related effects. Co-exposure, altered bioaccessibility, internal exposure, and toxicity should therefore be considered distinct levels of evidence. A key unresolved question is whether MP–metal co-exposure alters the internal metal dose and contributes to adverse health outcomes in humans.

Across trophic levels, biological responses to MP–metal co-exposure vary with taxa, polymer type, metal form, exposure route, and experimental conditions. Evidence is more informative when biological endpoints are accompanied by measurements of internal metal burden, bioavailability, or dietary transfer, but such measurements are absent from many studies. Both direct and dietary co-exposure can produce biological effects, yet current evidence does not support a consistent increase in metal transfer attributable to MPs across defined food chains. Broader interpretation remains limited by incomplete comparator groups, sparse concentration–response designs, inadequate assessment of depuration or retention, and the limited availability of quantitative prey–consumer transfer data.

## 6. Conclusions

Microplastic–metal interactions in aquatic environments are dynamic and depend on particle properties, metal speciation, and surrounding environmental conditions [17,21,26]. Adsorption and desorption can involve multiple interfacial processes, and their relative importance varies among MP–metal systems. Aging, biofilm colonization, particle size, and water chemistry can alter metal retention and release. Adsorption capacity alone is therefore insufficient to predict ecological consequences because retained metals may remain particle-associated or be redistributed after desorption under changing environmental or digestive conditions.

A trophic-level perspective highlights the need to distinguish the occurrence of MPs and metals in organisms from their transfer through connected food chains. Controlled feeding experiments demonstrate that MPs can move from prey to consumers, whereas field observations generally provide less direct evidence of prey-mediated transfer [20,58]. Evidence that MPs modify dietary metal transfer is strongest in a limited number of defined producer–consumer or prey–consumer systems in which metal uptake, retention, or bioaccessibility was compared between treatments [71,72]. Even in these studies, particle-bound transport cannot always be separated from changes in metal partitioning within prey or digestive mobilization. Current evidence therefore supports MP-mediated changes in metal transfer under some conditions but does not establish consistent particle-bound co-transport or biomagnification of metals across complex aquatic food webs.

Biological responses to MP–metal co-exposure are also context dependent. Both strengthened and attenuated responses have been reported, depending on the polymer properties, metal identity and concentration, exposure durations, species, and toxicological endpoints. Evidence is more informative when changes in metal accumulation or bioaccessibility are measured together with biological effects. A combined response that differs from either single-stressor treatment should not be classified as synergistic or antagonistic without comparison with an appropriate non-interaction reference model such as CA or IA [73,74,77]. Experimental gastrointestinal studies further indicate that MPs can modify metal release, bioaccessibility, or epithelial transport under some conditions [117,119]. Available human evidence, however, remains insufficient to determine whether these processes increase systemic metal exposure or contribute to specific adverse clinical outcomes.

The remaining uncertainties, limitations of the current evidence, and corresponding research priorities are summarized in Table 4. Resolving these issues will require stronger evidence linking MP–metal interactions to quantitative changes in metal transfer, retention, bioaccessibility, and toxicity across aquatic food webs.

## Figures and Tables

**Figure 1:**
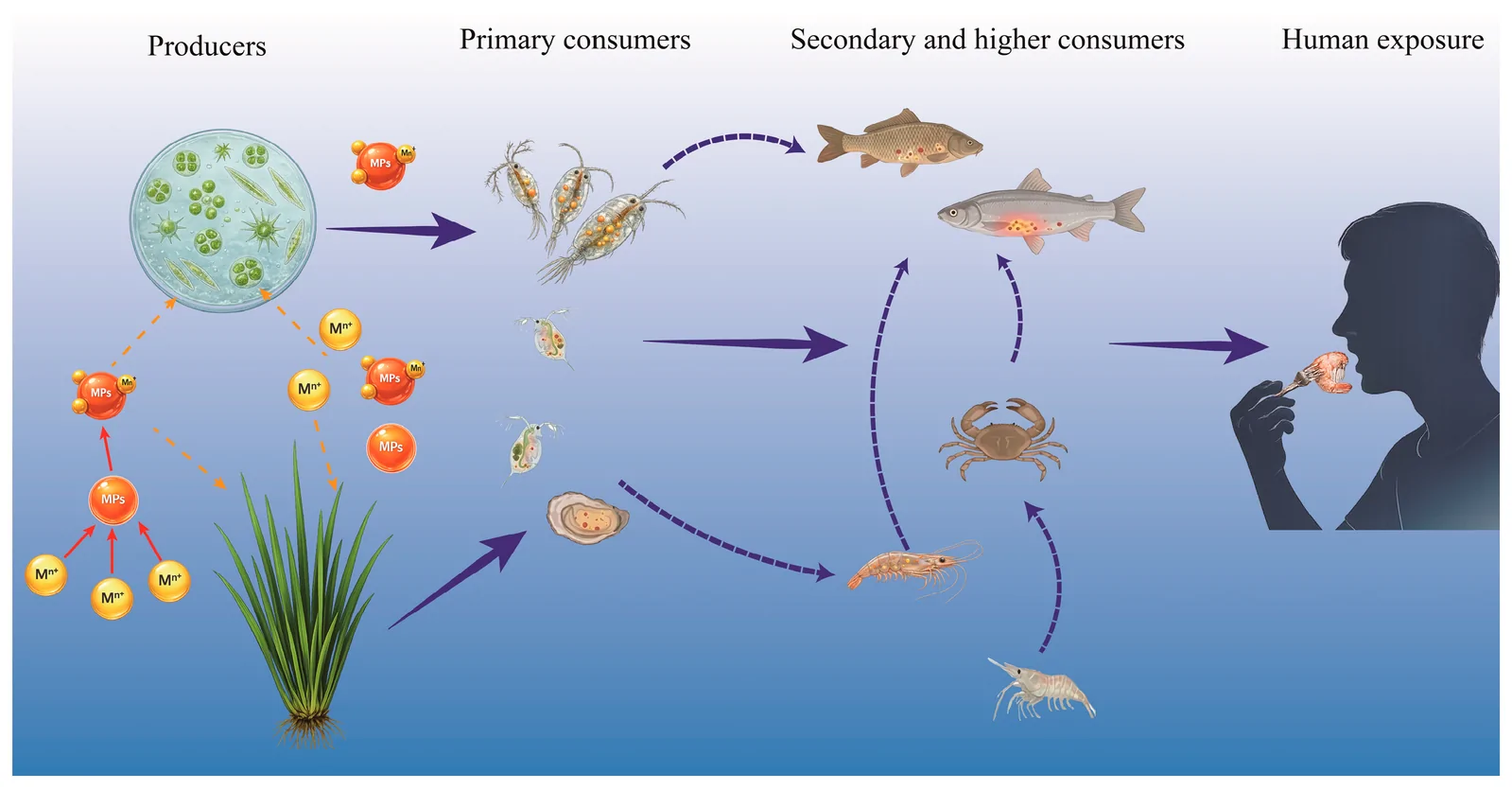
Transfer pathways of microplastics and heavy metals in aquatic food chains. Orange spheres labeled “MPs” represent microplastics, and yellow spheres labeled “M^n+^” represent dissolved metal ions. Red arrows indicate the adsorption of metal ions onto MPs. Orange dashed arrows indicate the exposure and uptake of MPs, dissolved metal ions, or metal-associated MPs by primary producers. Solid blue arrows show the overall direction of transfer across trophic levels, while blue dashed arrows show representative prey–consumer transfer pathways among aquatic organisms. The arrow leading to humans indicates potential dietary exposure through the consumption of aquatic organisms.

**Table 1:** Comparison of previous reviews on microplastic–metal interactions.

Review	Main Scope	Ecosystem Focus	Treatment of Trophic Transfer	Key Remaining Gaps
Cao et al. [17]	Sources of metals associated with MPs; adsorption kinetics, isotherms, and mechanisms; factors affecting adsorption; combined effects on organisms and humans	Aquatic and terrestrial environments	Food-chain transfer is discussed mainly as a potential ecological consequence and future research need	Does not systematically evaluate evidence that MPs modify metal transfer through defined aquatic prey–consumer links
Liu et al. [18]	MPs as vectors of metals; adsorption mechanisms and controlling factors; biological effects of MP–metal exposure	Aquatic environments	Trophic transfer is not a central analytical focus; transfer and accumulation of MP-associated metals are identified mainly as an important research need	Does not systematically connect MP–metal interfacial processes with measured dietary metal transfer across trophic levels
Adeleye et al. [19]	MP–metal interactions, combined effects, interaction mechanisms, and remediation strategies	Mainly aquatic environments	Trophic transfer is not a major focus	Emphasizes interaction mechanisms and remediation rather than evidence from prey–consumer transfer experiments
Huang et al. [20]	Ecotoxicological effects, combined effects, trophic transfer, and potential human health implications of MPs and associated contaminants	Aquatic environments	A major component of the review; includes prey–predator transfer and aquatic food-chain studies	Heavy metals are only one class among multiple MP-associated contaminants; MP-mediated metal-specific transfer is not evaluated as a distinct evidence question
Rasool et al. [21]	Quantitative and mechanistic analysis of MP–metal adsorption/desorption, environmental factors, toxicity, modeling, and remediation	Aquatic environments	Food-web transport is mentioned in relation to desorption and bioavailability, but is not the central evidence-synthesis framework	Focuses mainly on sorption/desorption and mitigation rather than direct comparison of metal-transfer evidence across prey–consumer pathways
Wang et al. [22]	Sources, co-exposure, bioaccumulation, and ecotoxicological risks of Hg and MPs	Aquatic environments and food webs	Food-web bioaccumulation is an important component	Restricted to Hg and does not provide a cross-metal assessment of MP-mediated changes in transfer through defined prey–consumer links

**Table 2:** Comparison of toxic effects induced by single and combined exposure to microplastics and heavy metals in aquatic organisms.

Organism	Exposure Conditions	Biological Effects	Mixture-Effect Assessment	Ref
*Vallisneria natans*	Fragmented PA MPs (78.16 ± 0.10 μm), 0.1%, 0.3%, or 1.0% (*w*/*w* sediment); Cd^2+^, 0.3, or 1.0 mg L^−1^; individual exposure, 14 d	At 1.0 mg L^−1^ Cd, plant height and RGR decreased by 49.63% and 65.25%, respectively. At 1.0% PA MPs, plant height decreased by 31.11%	Formal IA/MDR evaluation: synergism at PA 0.1% or 0.3% + Cd 0.3 mg L^−1^ (MDR 2.334 and 2.302); antagonism for all combinations containing Cd 1.0 mg L^−1^	[78]
	Same PA MPs combined with Cd; 14 d	At PA 0.3% + Cd 0.3 mg L^−1^, observed plant-height inhibition was 36.3%, compared with an IA-predicted value of 15.8%. At PA 1.0% + Cd 1.0 mg L^−1^, observed inhibition was 4.4%, compared with 65.3% predicted		
*Scenedesmus armatus*; *Microcystis aeruginosa*	PE microspheres (250–300 μm), 1 mg mL^−1^; Ag(I), 0.001 mg L^−1^; Cu(II), 0.05 mg L^−1^; or Cr(VI), 0.5 mg L^−1^; individual exposure, 28 d	In *S. armatus*, maximum growth inhibition was 10.91%, 4.91%, and 19.64% for Ag, Cu, and Cr, respectively. In *M. aeruginosa*, Cr exposure produced up to 64.21% growth inhibition	No formal CA/IA evaluation; responses were assessed by treatment comparisons and ANOVA.	[79]
	Same PE MPs combined separately with Ag(I), Cu(II), or Cr(VI); 28 d	In *S. armatus*, maximum growth inhibition was 1.48%, 5.12%, and 21.54% for Ag/MP, Cu/MP, and Cr/MP. In *M. aeruginosa*, Cr/MP produced 64.08% inhibition, similar to Cr alone		
*Brachionus koreanus*	PS NPs (~40 nm); ZnO NPs (~50 nm) or Zn ions; individual exposure; 7 d reproduction test	All three stressors inhibited reproduction in a concentration-dependent manner; individual toxicity followed PS NPs < ZnO NPs < Zn ions	Formal CA/IA/MDR and RS evaluation: PS NPs + ZnO NPs was antagonistic overall (CA MDR 0.077; IA MDR 0.094); PS NPs + Zn ions was additive under CA and IA (MDR 0.917 and 0.926)	[80]
	PS NPs (1, 10, or 20 mg L^−1^) combined with ZnO NPs or Zn ions; 7 d	PS NPs + ZnO-NP mixtures showed substantially lower toxicity than CA/IA predictions at higher PS NPs concentrations, whereas PS NPs + Zn-ion responses remained close to additive predictions		
*Daphnia magna*	Spherical PVC MPs (500 nm), 1 mg L^−1^; Cd, 50–675 μg L^−1^ for 48 h or 0.5 μg L^−1^ for 21/46 d; individual exposure	The 48 h EC_50_ of Cd was 215.3 μg L^−1^. Cd also impaired locomotion and induced DNA damage and oxidative stress, whereas PVC alone caused no marked acute toxicity	No formal CA/IA evaluation; main and statistical interaction effects were evaluated by two-way ANOVA	[81]
	PVC 1 mg L^−1^ + corresponding Cd exposure; 48 h, 21 d, or 46 d	PVC increased the Cd 48 h EC_50_ from 215.3 to 343.4 μg L^−1^ and attenuated acute Cd-induced locomotor, DNA, and oxidative damage. Longer co-exposure impaired development, reproduction, metabolism, intestinal structure, and mitochondrial function		
*Litopenaeus vannamei*	Spherical PE MPs (53–63 μm), 300 mg kg^−1^ feed; Cd, 30 mg kg^−1^ feed; individual dietary exposure, 21 d	Final weight was 4.2 ± 0.2 g with PE MPs and 4.1 ± 0.1 g with Cd, compared with 4.3 ± 0.2 g in controls; neither single exposure significantly affected growth or survival	No formal CA/IA evaluation; combined effects were determined by treatment-group comparisons	[82]
	PE MPs 300 mg kg^−1^ + Cd 30 mg kg^−1^ feed; 21 d	Final weight decreased to 3.8 ± 0.1 g. Hepatopancreatic Cd reached 91.7 ± 1.4 μg g^−1^, compared with 80.5 ± 5.6 μg g^−1^ under Cd alone (13.9% increase)		
*Channa punctatus*	Irregular PVC MPs (~0.315 μm), 0.5 mg L^−1^; Cu^2+^, 0.85 mg L^−1^; individual exposure, 60 d	At day 60, gut Cu reached 5.94-fold of control under Cu exposure. ROS increased 6.32-fold with PVC MPs and 5.82-fold with Cu alone	No formal CA/IA evaluation; stronger combined responses were based on treatment comparisons and two-way ANOVA	[83]
	PVC MPs 0.5 mg L^−1^ + Cu^2+^ 0.85 mg L^−1^; 60 d	Gut Cu accumulation reached 9.64-fold of control. GSH decreased to 0.42-fold, whereas LPO increased to 2.98-fold at day 60. Co-exposure produced the greatest Cu accumulation, oxidative stress, intestinal injury, and activation of cuproptosis- and PANoptosis-related pathways		

**Abbreviations:** CA: concentration addition; EC_50_: median effective concentration; GSH: reduced glutathione; IA: independent action; LPO: lipid peroxidation; MDR: model deviation ratio; PA: polyamide; PE: polyethylene; PS NPs: polystyrene nanoplastics; PS: polystyrene; PVC: polyvinyl chloride; RGR: relative growth rate; ROS: reactive oxygen species; RS: response surface; ZnO NPs: zinc oxide nanoparticles; ANOVA: analysis of variance.

**Table 3:** Trophic transfer of microplastics and metals in aquatic food chains.

**A. MP-Only Trophic Transfer**					
**Transfer Pathway**	**Exposure Design**	**Transfer and Accumulation**	**Biological Effects**	**Key Limitation**	**Ref**
*Bellamya aeruginosa* → *Mylopharyngodon piceus*	PS (100 nm or 10 μm) 1 mg L^−1^; snails were exposed for 12 h and then fed to fish once daily for 5 weeks; control fish received unexposed prey	Both particle sizes were detected in the gastrointestinal tract and muscle; tissue-specific transfer ratios were calculated; fish tissue concentrations continued to increase during exposure	Dietary transfer of 100 nm PS altered the fish gut microbiota: Proteobacteria increased by 14.18%, whereas Firmicutes and Bacteroidetes decreased by 9.57% and 3.53%, respectively. The potentially pathogenic genus *Plesiomonas* increased from 0.011 ± 0.00004% in controls to 0.16 ± 0.0007% and 0.14 ± 0.028% after 10 μm and 100 nm PS exposure	Demonstrates prey-to-consumer MP transfer but provides no evidence of metal transfer. Elimination rate constants were not measured; simplified two-level food chain	[60]
*Artemia salina* → *Litopenaeus vannamei* → *Oreochromis niloticus*	PS (1 μm) 1 × 10^6^ particles mL^−1^; *A. salina* were exposed for 24 or 48 h and subsequently fed to shrimp, which were then fed to tilapia; an unexposed food-chain control was included	MPs were detected mainly in the gut, intestinal epithelium, and feces; no detectable translocation to other tissues; reported BCF/BAF values < 1	Dietary MP transfer caused intestinal histological alterations and stress responses. In shrimp after 24 h, GPx, cMnSOD, and proPO expression increased 2.55-, 4.72-, and 8.39-fold, respectively. In tilapia after 48 h, SOD3, GPx, and IL-1β increased 4.4-, 1.4-, and 1.86-fold	Demonstrates transfer across three trophic levels but not MP–metal interaction or progressive accumulation. High particle concentration and short exposure limit environmental relevance	[64]
*Daphnia magna* → *Danio rerio*	PE (10–20 μm); zebrafish received 0.1 mg L^−1^ waterborne PE, clean prey, *D. magna* pre-exposed to 1 mg L^−1^ PE for 1 h or combined waterborne + trophic exposure; fish were exposed for 6 or 12 h	Dietary exposure produced greater MP uptake than waterborne exposure; 87.86–98.22% of detected particles were recovered in feces after 12 h in food-supplied groups	At the prey level, exposure to 25 mg L^−1^ PE reduced the feeding rate of *D. magna* by an average of 61.54%. No *Daphnia* mortality was observed, and no mortality or abnormal behavior was recorded in zebrafish during the short-term exposure	Particle transfer only. Shows that dietary exposure can dominate short-term uptake, but rapid elimination suggests limited retention	[65]
**B. MP–Metal Trophic Transfer**					
**Transfer Pathway**	**Exposure Design**	**Transfer and Accumulation**	**Biological Effects**	**Key Limitation**	**Ref**
*Paramecium caudatum* → *Danio rerio*	PS (2.14 μm), 1 × 10^5^ particles L^−1^ + Cd^2+^ (0.001, 0.01, or 0.1 μg L^−1^); *P. caudatum* exposed for 48 h, washed three times, and fed to zebrafish for 96 h; comparators: blank and 0.1 μg L^−1^ Cd^2+^	MPs and Cd were detected in zebrafish after prey-mediated exposure; no BMF or transfer efficiency was reported	At 0.1 μg L^−1^ Cd^2+^, liver size decreased from 0.055 ± 0.0075 μm^2^ with Cd alone to 0.048 ± 0.0077 μm^2^ with PS + Cd. Metabolic disturbance, gut microbiota dysbiosis, inflammation, DNA damage, and apoptosis were also observed	No MP-only trophic group; MP–Cd association during transfer was not directly tracked	[69]
*Chlorella vulgaris* → *Daphnia magna*	PS (2.0–2.9 μm), 0 or 15 mg L^−1^ + ZnO NPs (30 ± 10 nm), 0.5–8 mg L^−1^ for acute exposure; algae exposed for 48 h, washed, and fed to *D. magna* for 72 h. Chronic exposure used 0.5 mg L^−1^ ZnO NPs with 0 or 15 mg L^−1^ PS for 21 d	PS increased dietary Zn transfer. Acute BMF reached 1.49 with PS + ZnO but remained < 0.90 with ZnO alone; chronic BMF reached 2.11. At day 21, Zn in *D. magna* increased from 1.61 ± 0.10 to 3.10 ± 0.42 mg g^−1^ with PS	Compared with ZnO alone, PS + ZnO increased heart rate by 21.25%, ROS by 16.32%, SOD activity by 13.60%, and MDA by 7.31%. Intestinal ultrastructural damage, lipid-droplet accumulation, and growth inhibition were also enhanced	PS was removed before feeding; the study supports MP-mediated alteration of dietary Zn transfer rather than direct particle-bound MP–Zn co-transport. Total Zn did not distinguish particulate ZnO from released Zn^2+^	[71]
*C. vulgaris* → *D. magna*	PS (2.0–2.9 μm), 15 mg L^−1^ + ZnO NPs (30 ± 10 nm), 0.5 mg L^−1^; algae exposed for 48 h and PS removed before feeding; *D. magna* fed contaminated algae for 21 d	Although algal Zn was lower after PS + ZnO exposure (2.06 vs. 2.49 mg g^−1^), intestinal Zn in *D. magna* reached 1.09 mg g^−1^, 1.70-fold that of the ZnO-only group, indicating enhanced dietary Zn bioaccessibility and intestinal uptake	PS intensified ZnO-induced damage to algal prey, including increased membrane permeability, cell-wall rupture, and structural shedding, which was associated with greater digestive Zn release and subsequent intestinal uptake in *D. magna*	The mechanism is indirect and prey-mediated; PS was absent during consumer exposure. Particulate ZnO and dissolved Zn^2+^ contributions were not resolved	[72]
*P. caudatum* → *D. rerio*	PS (2.14 μm), 1 × 10^5^ particles L^−1^ + Cd^2+^, 0.001 μg L^−1^; *P. caudatum* exposed for 48 h, washed three times, and supplied to zebrafish twice daily for 90 d	Long-term prey-mediated transfer resulted in an estimated zebrafish Cd concentration of approximately 0.03 μg L^−1^ after 90 d. BMF and matched prey–consumer Cd burdens were not reported	Combined trophic exposure caused developmental abnormalities in the heart, liver, and gonads. Hepatic growth hormone decreased to 2.36 mU L^−1^, 45.1% below the control, with disruption of Wnt/β-catenin, growth-hormone, estrogen, and androgen signaling	No MP-only or Cd-only groups; the individual contributions of MPs and Cd, and any MP-mediated change in Cd transfer or toxicity cannot be separated	[84]

**Abbreviations:** BAF: bioaccumulation factor; BCF: bioconcentration factor; BMF: biomagnification factor; cMnSOD: mitochondrial superoxide dismutase; GPx: glutathione peroxidase; IL-1β: interleukin-1 beta; MDA: malondialdehyde; MPs: microplastics; PE: polyethylene; proPO: prophenoloxidase; PS: polystyrene; ROS: reactive oxygen species; SOD: superoxide dismutase; SOD3: superoxide dismutase 3; ZnO NPs: zinc oxide nanoparticles.

**Table 4:** Evidence gaps and future research priorities.

Remaining Uncertainty	Evidence Limitations	Future Research Priorities
Persistence and biomagnification of trophically transferred MPs	Trophic transfer has been demonstrated in controlled prey–consumer systems, but many studies are short term and lack adequate depuration, matched prey–consumer burdens, or quantitative transfer metrics. Transient ingestion therefore remains difficult to distinguish from persistent retention and biomagnification.	Longer-term feeding and depuration experiments; matched prey–consumer measurements; uptake and elimination kinetics; transfer efficiency and BMF assessment where applicable.
Persistence of MP–metal association during trophic transfer	Detection of MPs and metals in the same consumer does not establish particle-bound co-transport. MP–metal associations are rarely followed through ingestion and digestion, while total metal measurements often cannot distinguish dissolved, particulate, and MP-associated fractions.	Simultaneous tracking of MPs and associated metals across prey, digestive phases, and consumers; metal-speciation analysis; contaminant mass-balance approaches.
Contribution of MPs to changes in metal toxicity	Stronger or weaker responses under co-exposure do not necessarily reflect MP-mediated changes in metal exposure. Incomplete single-stressor controls, limited internal-metal measurements, and the absence of formal CA or IA evaluation restrict interpretation of mixture interactions.	Complete single- and combined-exposure designs; concurrent measurement of internal metal burdens and biological responses; concentration–response and mixture-ratio designs; CA or IA evaluation where interaction classification is intended.
Environmental relevance and generalizability of laboratory evidence	Many experiments rely on pristine particles, simplified trophic links, relatively high concentrations, and controlled water matrices. Particle aging, biofilm formation, water chemistry, metal speciation, and species-specific feeding behavior can substantially modify MP–metal interactions.	Greater use of environmentally relevant concentrations and weathered particles; incorporation of biofilms and realistic water chemistry; better particle and metal characterization; longer-term and more complex food-web experiments.

## Data Availability

No new data were created or analyzed in this study. Data sharing is not applicable to this article.
